# Notes and News

**Published:** 1889-02-02

**Authors:** 


### Notes and News.

Collected and Communicated.

The Tiverton Infirmary and Dispensary is able to report that it has done more work last year than ever previously, and has done it more cheaply. The new rules and new matron are evidently satisfactory. Over a thousand patients were treated last year.

The annual meeting of the subscribers to Dover Hospital was graced this year by the presence of many ladies. The report submitted was satisfactory: In-patients, 123; out-patients, 3,392. Balance in hand, £657. Mr. Wood was elected honorary surgeon in place of Dr. Marshall, resigned.

The Montefiore Home for Chronic Invalids, New York, was opened on December 18th. It is a beautiful building, and can accommodate 180 patients. Though built by Jews especially to supply the needs of those of their own faith, it is not sectarian; its doors are open to all sufferers.

A committee is being formed to promote the establishment of a School of Ambulance at Woolwich, as a memorial to the late Colonel Duncan. It is further proposed to place a memorial brass in the garrison church at Woolwich, and to erect a bust, executed during life, in Holborn, the borough represented by the gallant gentleman in Parliament.

Christmas was kept rather late at Carmarthen Infirmary, but it was well kept all the same. Miss Macintyre and the medical staff arranged an attractive programme, and help and contributions were given liberally by outsiders. The occasion drew together many who have not hitherto taken a great interest in the infirmary, and it is to be hoped that the pretty sight witnessed on January 10th will lead many to help in extending the usefulness of this excellent institution.

The annual meeting last week of the General Council of the Great Northern Central Hospital had a sad, yet satisfactory, tale to hear. In spite of the large new buildings, the hospital has not room for all who apply for admittance, and it does a great and good work, but would fain do a greater. Three thousand accidents were treated last year, so the hospital has apparently arisen on a site where its presence was needed. Finances are satisfactory, for the visit of the Prince of Wales in July, and the excitement of opening the new building, has kept the Great Northern well before the public. The Islington Jubilee Fund, of over £5,000, has also been a special aid. A committee of ladies, led by the Baroness Burdett-Coutts, are working hard for the hospital, and doubtless 1889 will see another £20,000 raised to complete the handsome edifice, and open sufficient accommodation to meet the needs of the neighbourhood. Prince Albert Victor of Wales has been appointed president.

The question of the extension of Aberdeen Infirmary is again to the fore, for the committee find they cannot erect a building to hold 240 beds for £25,000. The committee have an excellent scheme before them, and contemplate all the latest improvements, such as a slate dado like that in the Leith Hospital. But such a building as they desire will cost at least £50,000, and it might be better to build a wing at a time, and not run into debt.

On January 23rd Sir Evelyn Baring opened the new wing of the Victoria Deaconesses Hospital, Cairo, in the presence of a large attendance of all nationalities. The new wing is for the treatment of infectious and mental cases, for which there has formerly been no provision at Cairo. Sir E. Baring thanked the foreigners for their presence, he also referred to Lady Baring's late recovery from a serious illness, attributing her present wellbeing to the excellent nursing of one of the nurses from the Jubilee Institute at Alexandria.

On January 22nd was opened the new Jubilee Hospital, at Glossop, erected and endowed by the late D. Wood, Esq. Owing to the recent death of the generous donor, there was no ceremony connected with the opening, but it is hoped that when the Prince of Wales is at Buxton next summer he will be prevailed upon to visit the hospital and the other Jubilee gifts to the town. The building is in the shape of an E, and contains two wards 40ft. by 24ft., one for men and one for women. The architect is Mr. Murgatroyd, of Manchester.

On December 17th the annual meeting was held of the Norwich Hospital Sunday Fund. The income amounted to £935, as compared with £941, but the working expenses had been reduced £4, so that the difference really only reached £1. It was proposed in future to readjust the percentage given to various charities; to decrease the sum awarded to the Norfolk and Norwich Hospital, and the Hospital Staff of Nurses; and increase the sum awarded to the Lowestoft Convalescent Home, the Lying-in Charity, and the Dispensary.

The Mayor of Nottingham, when presiding at the annual meeting of the Hospital for Women last week, had the courage to depart from conventional compliments, and advised certain improvements in the management of the hospital. The notion that charities are not to be criticised is one which we are glad to say is fast disappearing; and with it will also disappear those scandals which now sometimes shock the philanthropic world. He who advocates reform must be prepared for much abuse, and must find for himself his own reward.

At the late quarterly meeting of the Norfolk and Norwich Hospital Committee, the chairman spoke of several improvements in the system of management, which are to be carried out before the annual report is issued. At present the rules for the lady superintendent, house surgeon, and others, overlap and interfere with one another. The dispenser is also to teach dispensing in future, and a nurse is to be sent to London to learn massage. It is also proposed to appoint an honorary dental surgeon. The revised report, containing particulars of all these improvements, will be looked for with interest.

As is customary at Inverness and other northern towns, the first Sunday of the year was devoted to Hospital Sunday. At Inverness, sermons in behalf of the Northern Infirmary were preached in nearly every church, and some large collections were made. The ministers were able to make a spirited appeal to their congregations this year, for they had nearly all been present at the impressive opening ceremony of the enlarged infirmary only a few days before. Treating 387 in-patients yearly, the Northern Infirmary gathers in the sick of the whole of the North of Scotland. The work it does is a good one, and worthy of all support.

The following vacancies are announced this week, the amount of salary in each case being given after the name of the institution :—

**Resident Medical Officers.**

Birmingham General Hospital. Assistant House Surgeon.—Board, etc.
Kilmallock Union. Medical Officer.—£178.
Northern Infirmary, Inverness. House Surgeon.—£50, board, etc.
Parish of St. George's-in-the East. Assistant Medical Officer.—£120, board, etc.
South-Eastern Fever Hospital. Clinical Assistant —Board, etc.
Friends' Retreat, York. Junior Assistant Medical Officer.—£80, board.

**Non-resident Medical Officers.**

Sussex County Hospital. Physician and Assistant Physician.
Dental Hospital of London. Dental Surgeon.

---

The annual meeting of the Charity Organisation Society last week was more interesting than usual. Twice the subject of medical charities was touched on, and Lord Balfour of Burleigh spoke strongly on the necessity of trying to exclude those who are not necessitous from our hospitals and infirmaries. The annual report was adopted, and it was resolved that greater use should be made of local centres. The objects of the society are the prevention of pauperism and the prevention of the abuse of charity. To secure this it affiliates itself with existing relief agencies, and makes inquiries into all doubtful cases, trying to render aid in such a manner as to draw out the energy of the applicant, and teach him independence once more. The society always welcomes either personal or pecuniary help from those who appreciate its methods.

---

The annual entertainment to the patients in the King's College Hospital was held on January 24th in the large hall, which was decorated by the students with festoons of evergreens, flags, and shields. In one corner a stage, with footlights, curtain, and furniture complete, was erected for the convenience of the "Jamboree Niggers," and for that of the performers who had kindly volunteered their services. For many years past Mr. Toole has rarely been absent from these entertainments ; this year, however, Mr. Toole was unable to appear, because of a slight congestion of the throat. Mr. Lonnen, ready dressed for his part of Mephistopheles, sang his comical song, and Mr. Collette and Miss Mary Collette gave "Collette at Home." Those of the patients who were well enough to be moved were wheeled into the corridors, from which they could command a good view of the stage.

---

At a meeting of the council of the Metropolitan Hospital Sunday Fund, under the presidency of the Right Hon. James Whitehead (the Lord Mayor), arrangements were made to organise the collections on the Stock Exchange, Coal Exchange, Metal Market, and elsewhere this year. A suggestion to make a house-to-house collection was not adopted, but one to make the distribution of surgical appliances more general was favourably received and referred to the General Purposes Committee. Sir Sydney Waterlow, Bart., announced that after an explanation as to the expenses of the annual dinner, the Distribution Committee had determined to increase the grant to the North London Consumption Hospital, which, thanks to the chairman (Mr. B. A. Lyon) and his colleagues, is now one of the best-managed of London hospitals. In the discussion which followed it was pointed out that whereas there is much to be said against holding an annual dinner in support of an old-established charity, where the institution is comparatively young, and especially after being reorganised, the dinner is not only a legitimate, but a fruitful source of income. The proposal to increase the grant was unanimously adopted, and we congratulate the committee of the North London Consumption Hospital, who have well earned such a genuine compliment, which ought to have a material influence on the resources placed at their disposal by the public.

The Rev. M. Comardy, who has gone to Molokai to take the place of the dying Father Damien, writes a pathetic account of the leper settlement. He says :—"The poor Father has suffered dreadfully. He is completely disfigured ; his voice is almost extinct. If you could only see him as he lies in his little room, on the floor, upon his bed of suffering, tears would come into your eyes at the sight of that man who has done so much for thousands of lepers, now himself reduced to so terrible a condition, and so very little can be done for him. People call it a sacrifice to live with lepers ; but only on seeing oneself a leper, and nothing but lepers around, then only does the extent of the sacrifice become apparent." The age of heroes and martyrs is not yet passed.

---

Changes have been numerous at the Royal Eye Infirmary, Plymouth, during the last year, and at the annual meeting last week many well-known faces were absent. Miss Meeres has been appointed matron in the place of the late Mrs. Black, and the late dispenser (Mr. Boyntun) died on August 23rd last. A change in the medical staff has taken place by the resignation of Mr. Eccles, one of the surgeons, who for more than thirty-two years had given his valuable aid and skill, but he will continue to render his advice, when needed, in the capacity of consulting surgeon in the place of Mr. W. G. Square, who had resigned in his favour. Mr. Square has been elected a life member of the committee. Mr. G. Elliot Square has filled the vacancy, being elected at a special meeting in December last.

---

The meeting at the Mansion House on Tuesday, under the auspices of the Hospital Saturday Fund, at which Mr. Coote explained the penny-a-week movement, was largely attended. It affirmed the principle, and referred the scheme to the Council of the Hospital Saturday Fund, to devise methods to secure a satisfactory response. A representative working man, Mr. Sainsbury, and Mrs. Thompson Weir, a district visitor, pointed out that unless the basis on which subscriptions were to be sought and received was first formulated and made public, the working classes would not contribute more generally than at present to the fund Mr. Burdett endorsed these contentions, and justified them by quoting the experience of the provincial towns where the penny-a-week movement had proved successful, because all classes were adequately represented on the organizing committee, which invariably formulated the basis before asking for the pennies. The Lord Mayor (the Right Hon. James Whitehead) grasped the situation at once, and desiring to make the scheme a success, arranged with those on the platform that it should be referred to a special committee of thirty-one, with power to add to their number, consisting of ten members elected by the Hospital Sunday Council, ten members elected by the Hospital Saturday Fund, ten representatives of the hospitals, firms, and working classes, with the Lord Mayor as chairman. Mr. Bunn, the chairman of the Hospital Saturday Fund Council, refused, however, to agree to any such arrangement, and as Mr. Burdett declined to move his proposal as an amendment, because success could only be secured by the united action of all the interests concerned, the Lord Mayor having obtained a promise that the suggestion should be acted upon, Mr. Coote's proposals were referred to the Council of the Hospital Saturday Fund. It rests with that body, therefore, to decide whether the scheme shall be properly organised by being referred in the first instance to a representative committee, or whether they will risk failure, involving serious loss to the hospitals, by opposing the general wish for a joint committee, and refusing to first formulate a basis and then to ask for funds. We cannot believe they will, on reflection, refuse the joint committee, especially as the Lord Mayor has consented to act as its chairman, and so to give the movement a solidity and position which it must otherwise want.

